# Spleen-derived circulating intercellular adhesion molecule 2 promotes osteosclerosis in chronic myelofibrosis

**DOI:** 10.1038/s41413-026-00575-9

**Published:** 2026-09-20

**Authors:** Sirion Aksornthong, Kerstin Tiedemann, Mélanie Welman, Alexandra Mazharian, Marie Lordkipanidzé, Yotis A. Senis, Svetlana V. Komarova

**Affiliations:** 1 Shriners Hospital for Children, Montreal, QC Canada; 2 Department of Experimental Surgery, Faculty of Medicine and Health Sciences, McGill University, Montreal, QC Canada; 3 Faculty of Dental Medicine and Oral Health Sciences, McGill University, Montreal, QC Canada; 4 Research Center, Montreal Heart Institute, Montreal, QC Canada; 5 Centre for Cardiovascular and Nutrition Research, Institut National de la Santé et de la Recherche 1263, Institut National de Recherche pour l’agriculture, l’alimentation et l’environnement 1260, Faculty of Medicine, Aix-Marseille University, Marseille, France; 6 Faculty of Pharmacy, University of Montreal, Montreal, QC Canada; 7 Department of Biomedical Engineering, University of Alberta, Edmonton, AB Canada

**Keywords:** Pathogenesis, Cancer, Cancer

## Abstract

Mutations in the gene coding G6b-B, *Mpig6b* in mice, and *MPIG6B* in humans lead to myelofibrosis, progressive splenomegaly and osteosclerosis. Aberrant megakaryocyte function in bone marrow and spleen may affect bone cells through cell-cell contact or soluble factors. We aimed to assess the role of circulating factors produced by splenic cells in mediating osteosclerosis in *Mpig6b*^*−/−*^ mice using splenectomy, a procedure commonly used in clinics. Splenectomy or sham surgery was performed on 32-week-old female *Mpig6b*^*−/−*^ and littermate wildtype (WT) mice, when splenomegaly and long bone osteosclerosis were established. Twenty weeks later, the hematopoietic parameters and bone structure of the femur and lumbar vertebra were analyzed. Splenectomy significantly improved platelet count, but the larger platelet size persisted in *Mpig6b*^*−/−*^mice. The *Mpig6b*^*−/−*^ femurs with established osteosclerosis were not affected by splenectomy. In contrast, splenectomy prevented the development of osteosclerosis in vertebrae of *Mpig6b*^*−/−*^ mice. Significant vertebral bone loss and increased circulating TRACP 5b were demonstrated in splenectomized *Mpig6b*^*−/−*^ mice compared to sham-operated and WT mice. Using quantitative plasma proteomics, we identified intercellular adhesion molecule 2 (ICAM2) as significantly increased in *Mpig6b*^*−/−*^ mice compared to WT and reversed to normal levels after splenectomy. The interaction of membrane-bound ICAM2 and MAC1 was reported to be critical for osteoclast fusion. Our in vitro study revealed that soluble recombinant ICAM2 dose-dependently inhibited osteoclast fusion, suggesting that competitive binding of MAC1 with soluble ICAM2 prevented its interaction with membrane-bound ICAM2. Thus, we identified the soluble ICAM2 as a novel inhibitor of osteoclast fusion and mediator of osteosclerosis in *Mpig6b*^*−/−*^ mice.

**Figure:**
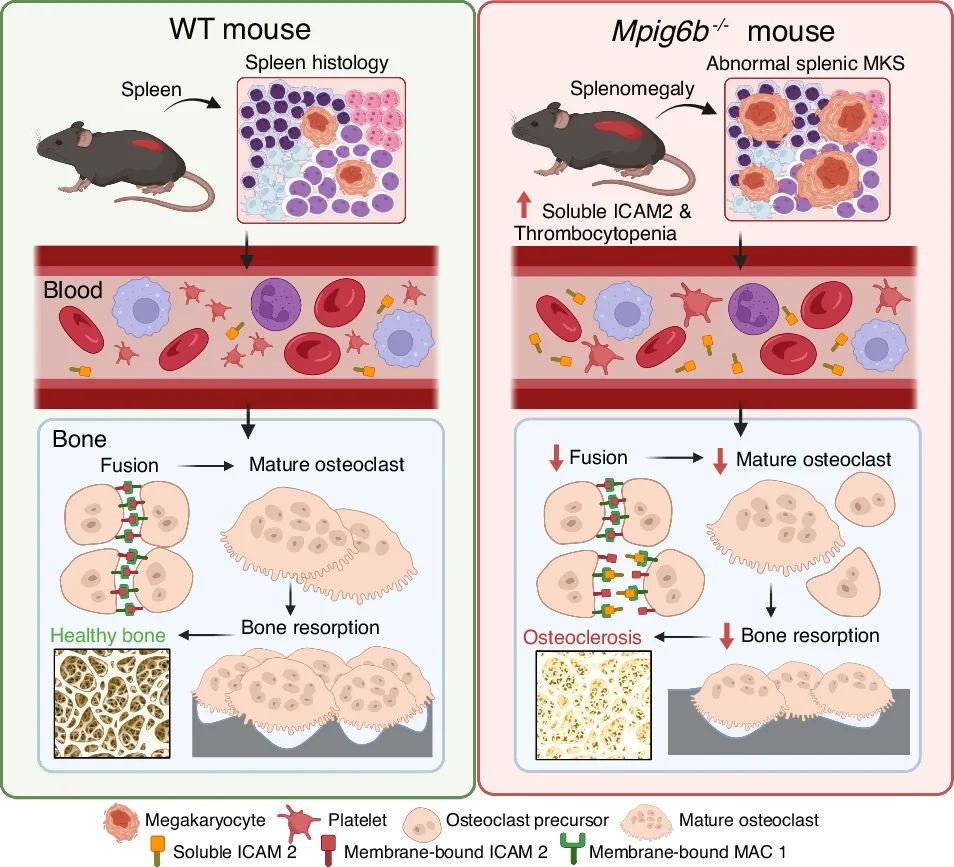

## Introduction

Primary myelofibrosis (MF) is a myeloproliferative neoplasm (MPN) characterized by bone marrow fibrosis, osteosclerosis, ineffective erythropoiesis, and extramedullary hematopoiesis in the spleen and liver, leading to hepatosplenomegaly.^1^ Myelofibrosis also arises as a complication of other MPNs, essential thrombocythemia, and polycythemia vera.^2,3^ Patients with MF have a 5-year relative survival of ~50% and present with many symptoms, including cachexia, fatigue, bone pain, thrombosis, and bleeding, which reduce their quality of life.^3–5^ Progressive splenomegaly is common in patients with advanced MF and is linked to debilitating symptoms such as abdominal pain, difficulty bending and walking, and weight loss.^6,7^ Surgical spleen removal, splenectomy, is used for treating massive splenomegaly in patients with MF, especially in cases of disease refractory to conventional drugs, and is reported to lead to improvements in anemia, thrombocytopenia, and portal hypertension.^8,9^ However, the link between splenomegaly and osteosclerosis is not well understood.

The development of osteosclerosis in MF has been linked to a dysregulation of MK proliferation and differentiation.^10^ Osteosclerosis has been demonstrated in multiple mouse models of MF, and MKs were shown to affect bone-forming osteoblasts and bone-resorbing osteoclasts via direct cell-cell interactions and soluble (circulating) factors.^11^ Transforming growth factor-β1 was shown to be essential for MF^12^ and a key contributor to osteosclerosis;^13^ however, other molecular players are also involved.^11,14,15^ Of interest, splenic MKs were suggested to be the source of secretory factors affecting bone in platelet-type von Willebrand disease mouse, which were shown to have normal bone marrow MKs, but high spleen MKs as well as a reduced osteoclast number.^16^ The nature and specific role of splenic MK-derived factors in mediating the progression of osteosclerosis is not known.

G6b-B is a megakaryocyte lineage-specific immunoreceptor tyrosine-based inhibition motif-containing receptor, which is a critical regulator of the megakaryocytic lineage, essential for platelet production and activation.^17^ Humans and mice with a deficiency in the gene coding for G6b-B, *Mpig6b* in mice and *MPIG6B* in humans, develop macrothrombocytopenia, large clusters of MKs in the bone marrow, extramedullary hematopoiesis, and MF in the marrow and spleen.^18–20^ We have shown that *Mpig6b* deficiency in the MK lineage leads to severe splenomegaly and progressive osteosclerosis in female *Mpig6b*^*−/−*^ mice, whereas male mice displayed adaptive bone resorption and delayed osteosclerosis. The higher bone formation and impaired bone resorption were demonstrated in *Mpig6b*^*−/−*^ mice.^21^ However, the pathogenesis of osteosclerosis in MF due to G6b-B deficiency remains unclear.

The aim of this study was to examine how splenectomy affects the development of osteosclerosis in *Mpig6b*^*−/−*^ mice. We hypothesized that the splenectomy in *Mpig6b*^*−/−*^ mice would reduce splenic cell-derived circulating factors and slow the progression of osteosclerosis. In this study, we used 32-week-old female *Mpig6b*^*−/−*^ mice, which had established osteosclerosis in their long bones, but the vertebra remained unaffected.^21^ This allowed us to assess the effect of splenectomy on preventing osteosclerosis development in vertebra and on reversing osteosclerosis in long bones.

## Results

**Fig. 1:**
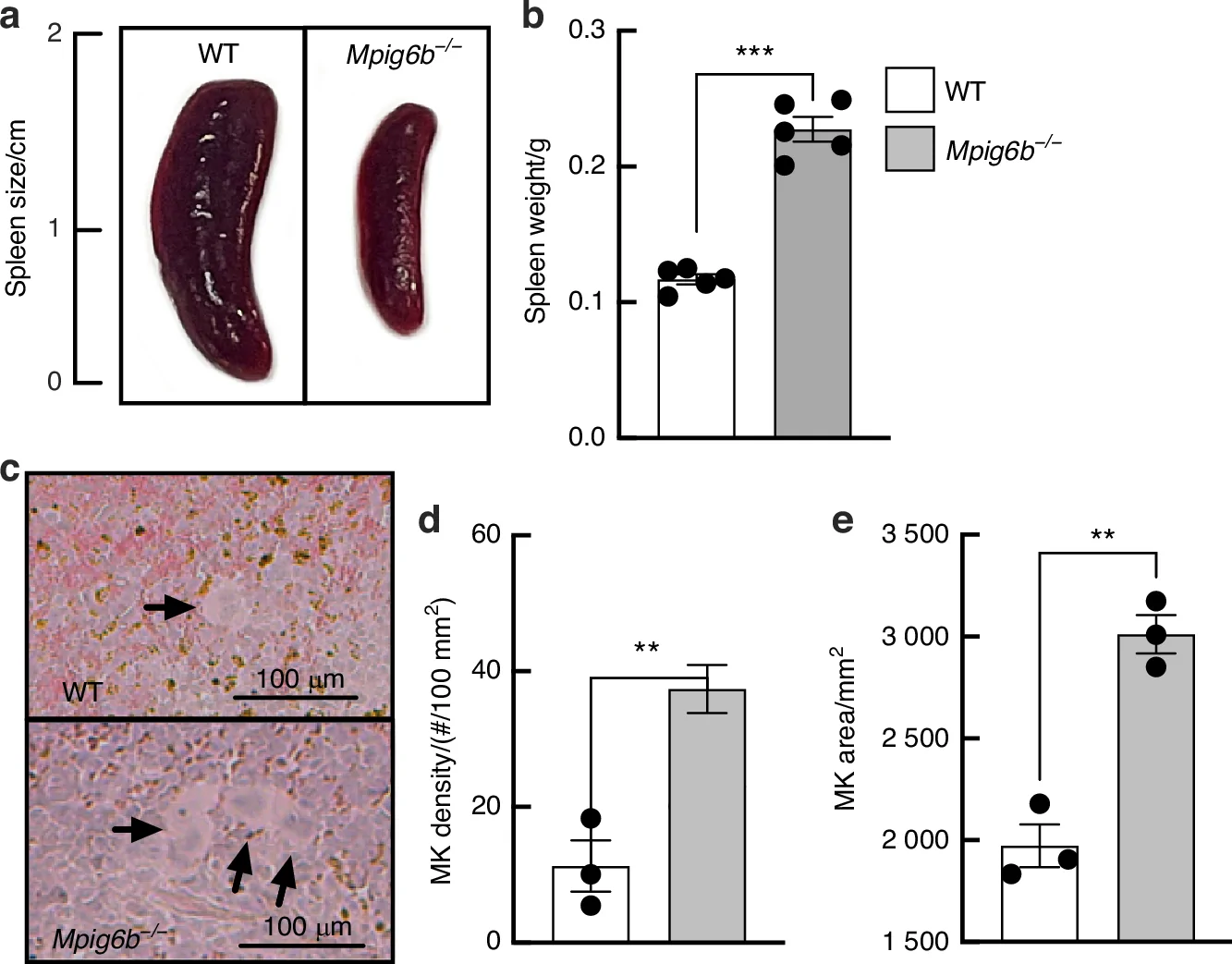
Spleens and splenic megakaryocytes in *Mpig6b*^−/−^ mice. Spleens were isolated from female 32-week-old WT (white bars) and *Mpig6b*^−/−^ (gray bars) mice. Representative pictures (**a**) and average weight (**b**) of spleens of WT and *Mpig6b*^−/−^ mice, *n* = 5 mice per group. **c** Representative pictures of H&E-stained sections of WT (top) and *Mpig6b*^−/−^ (bottom) spleens, arrows indicate megakaryocytes. Quantification of megakaryocyte density (**d**) and area (**e**) in the spleen section of WT and *Mpig6b*^−/−^, *n* = 3 mice per group (10 pictures per mouse). **P < 0.01, ***P < 0.001 by Student’s *t*-test

Splenomegaly is reported as a complication of progressive MF in humans,^22–24^ including patients with mutations in the *MPIG6B* gene.^25–27^ Moreover, we have previously identified osteomyelofibrosis and splenomegaly in female *Mpig6b*^*−/−*^ mice.^21^ We further confirmed that by 32 weeks of age, spleens were significantly enlarged in female *Mpig6b*^*−/−*^ compared with WT mice (Fig. 1a, b). In contrast, splenomegaly was not evident in male mice by 32 weeks of age.^21^ The histological analysis of spleens of 32-week-old female *Mpig6b*^*−/−*^ mice demonstrated the significantly increased MK density (Fig. 1c, d) and area (Fig. 1e) compared to WT mice. These data demonstrate that the splenomegaly in *Mpig6b*^*−/−*^ mice is associated with enhanced splenic megakaryopoiesis and abnormal MK morphology. The goal of this study was to examine whether and how abnormal spleen cells contribute to osteosclerosis in female *Mpig6b*^*−/−*^ mice. Splenectomies were performed in 32-week-old female *Mpig6b*^*−/−*^ and WT mice, while control animals were exposed to a sham operation, which included the same pre- and post-surgical steps, but spleens were not removed. The animals were followed for 20 weeks post-surgery.

**Fig. 2:**
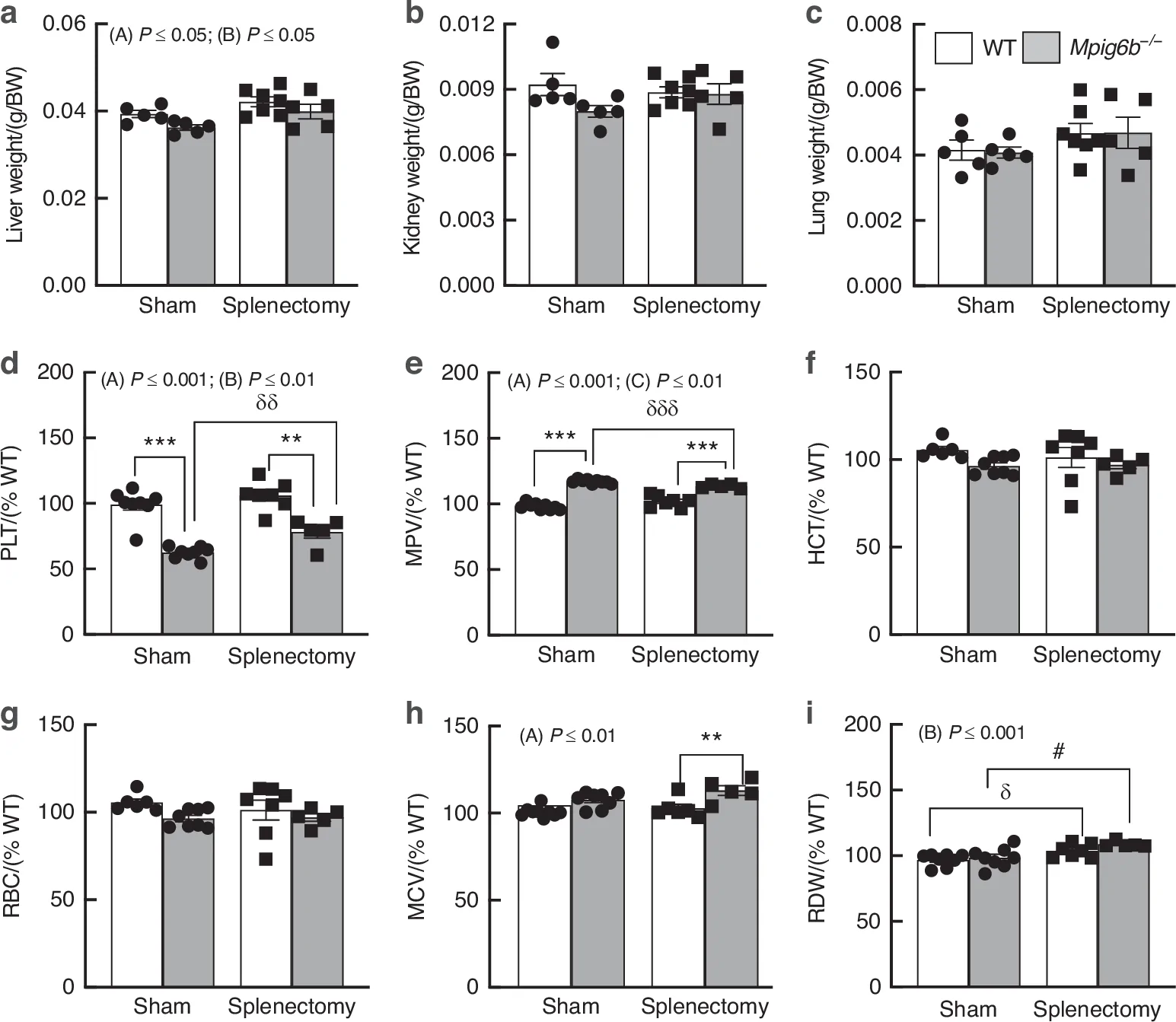
Effect of splenectomy on organ weights and hematological parameters in *Mpig6b*^−/−^ mice. Splenectomy or sham operations were performed in 32-week-old female WT (white) and *Mpig6b*^−/−^ (gray) mice, and 20 weeks later, organ weights and hematological parameters in whole blood were assessed. Average weights of liver (**a**), kidney (**b**), and lung (**c**) normalized per mouse weight. Whole blood analysis of **d** platelet counts, **e** platelet size, **f** hematocrit, **g** red blood cell (RBC) count, **h** mean corpuscular volume, and **i** RBC distribution width (RDW). *n* = 5–9 mice per group. Data were normalized with the average value in the WT group and presented as % of WT. Statistical analysis was performed by two-way ANOVA, main effects: **a** genotype effect, **b** surgery effect, and **c** interactions. Bonferroni multiple comparisons: **P < 0.01, ***P < 0.001 for comparing genotypes in the same surgery group and #*P* < 0.05 for comparing surgery groups in *Mpig6b*^−/−^mice. Unpaired Student’s *t*-test: was performed according to the significant effect of genotype reported in platelet counts (**d**) and platelet size (**e**), and the effect of surgery reported in RDW (**i**); ^δ^*P* < 0.05, ^δδ^*P* < 0.01, and ^δδδ^*P* < 0.001

First, we examined the impact of splenectomy on hematopoiesis 20 weeks after surgery. To assess potential sites of extramedullary hematopoiesis, we measured the weights of the liver (Fig. 2a), kidney (Fig. 2b), and lung (Fig. 2c), as these organs have previously been implicated in this process.^28–31^ Analysis by two-way ANOVA revealed significant effects of both genotype and surgery on liver weights. *Mpig6b*^*−/−*^ mice generally exhibited lower liver weights compared to WT controls (Fig. 2a). In contrast, kidney and lung weights did not differ significantly between groups (Fig. 2c). These findings indicate that, in this model, the liver, kidney, and lung are unlikely to participate in compensatory extramedullary hematopoiesis following splenectomy in *Mpig6b*^*−/−*^ mice.

Platelet and erythrocyte parameters were analyzed in whole blood samples. Consistent with previous reports, *Mpig6b*^*−/−*^ mice showed significantly lower platelet counts (Fig. 2d) and larger platelet size (Fig. 2e) than WT mice. While both platelet count and size improved following splenectomy in *Mpig6b*^*−/−*^ mice, these measures remained significantly lower than those of splenectomized WT mice. In contrast, there were no differences in hematocrit (Fig. 2f) and red blood cell (RBC) count (Fig. 2g) between *Mpig6b*^*−/−*^ and WT mice. However, mean corpuscular volume (MCV) demonstrated a significant genotype effect but was unaffected by surgery (Fig. 2h). Additionally, in line with known effects of splenectomy,^31^ RBC distribution width was significantly increased after splenectomy in both *Mpig6b*^*−/−*^ and WT mice (Fig. 2i). Altogether, these results indicate that splenectomy raised platelet count in *Mpig6b*^*−/−*^ mice, albeit to a lesser degree than in WT mice, with no meaningful changes in other blood parameters.

**Fig. 3:**
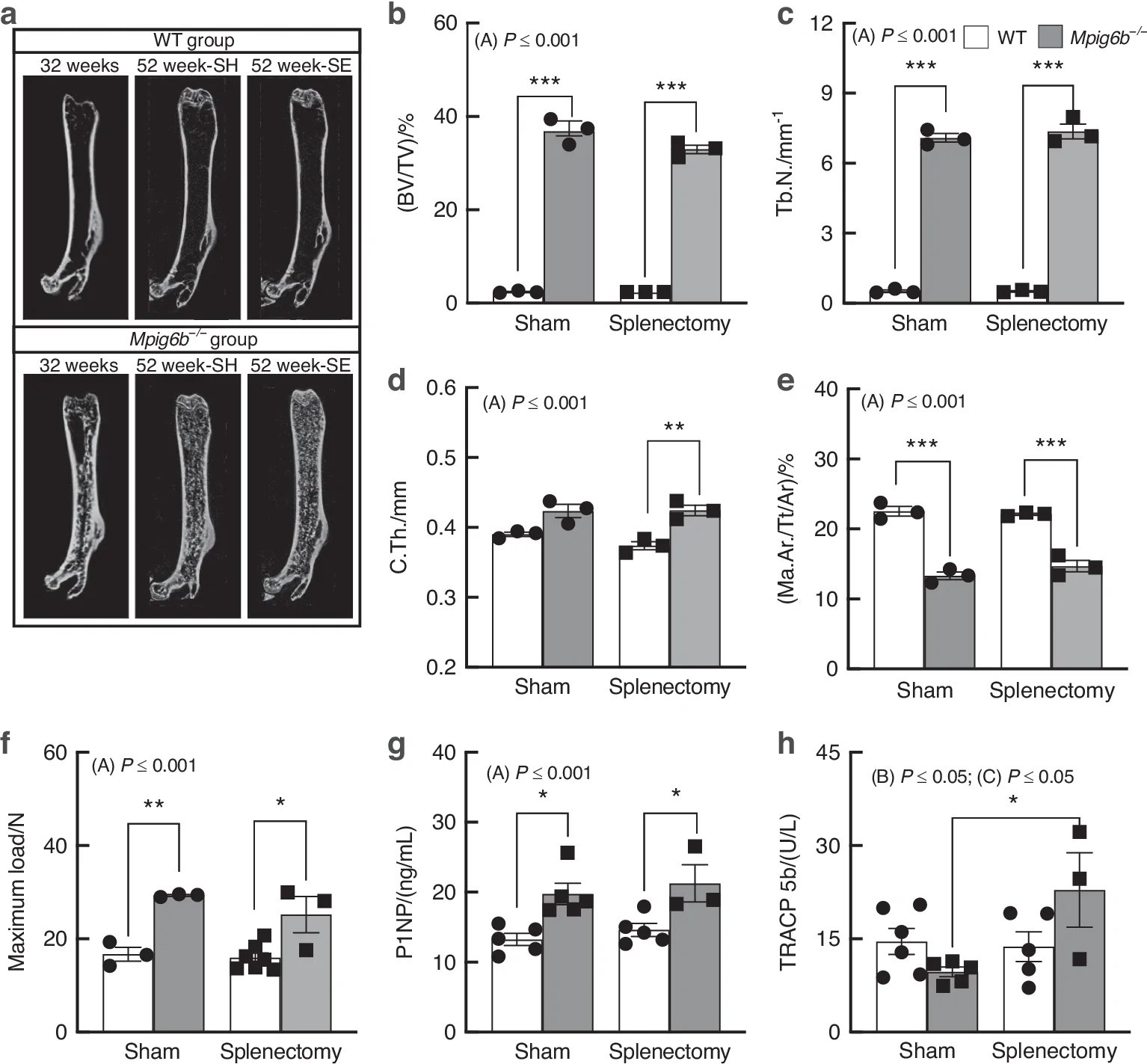
Effect of splenectomy on femurs in *Mpig6b*^−/−^ mice. Splenectomy or sham operations were performed in 32-week-old female WT (white) and *Mpig6b*^−/−^ (gray) mice, and femurs were characterized 20 weeks later. **a** Representative μCT reconstructions of the femur in WT and *Mpig6b*^−/−^ mice in the sham and splenectomy groups at the baseline (32 weeks of age) and 20 weeks post-surgery (52 weeks of age). Bone microarchitecture was examined by μCT, shown are mid-diaphysis values for **b** bone volume per tissue volume (BV/TV, %), **c** trabecular number (Tb.N, mm^−1^), **d** cortical thickness (C.Th., mm), and **e** bone marrow area per tissue area (Ma.Ar/T.Ar, %). **f** Maximum load (*N*) assessed by 3-point bending test of biomechanical properties. Analysis of bone turnover markers in plasma using **g** P1NP for bone formation marker (ng/mL) and **h** TRACP 5b for bone resorption marker (U/L). *n* = 3–7 mice per group, statistical analysis by two-way ANOVA, main effects: **a** genotype effect, **b** surgery effect, and **c** interactions. Bonferroni multiple comparisons: *P < 0.05, **P < 0.01, ***P < 0.001 for comparing genotypes in the same surgery group

Next, we evaluated the effect of splenectomy on bone microarchitecture and mechanical properties in long bones with pre-existing osteosclerosis.^21^ At the age of 32 weeks, female *Mpig6b*^*−/−*^ mice demonstrated femoral osteosclerosis that progressively worsened with increasing age. In 52-week-old female *Mpig6b*^*−/−*^ mice, femoral osteosclerosis was characterized by significantly increased bone volume/tissue volume (BV/TV; Fig. 3a, b), trabecular number (Fig. 3c), and cortical thickness (Fig. 3d), along with reduced bone marrow area/total cross-sectional area (Fig. 3e), compared to WT controls. These differences were observed in both sham-operated and splenectomized groups of *Mpig6b*^*−/−*^ mice. In addition, biomechanical analysis using a 3-point bending test showed femurs from *Mpig6b*^*−/−*^ mice withstood higher maximum loads than those from WT mice (Fig. 3f), regardless of surgical intervention. Plasma levels of bone turnover markers, P1NP for bone formation and TRACP 5b for bone resorption, were assessed. *Mpig6b*^*−/−*^ mice showed a significantly higher P1NP level in both sham-operated and splenectomized groups compared to WT mice (Fig. 3g), indicating that increased bone formation in *Mpig6b*^*−/−*^ mice was unaffected by splenectomy. Surprisingly, splenectomy led to a significant increase in plasma TRACP 5b level in *Mpig6b*^*−/−*^ mice, indicating higher bone resorption activity post-splenectomy specifically in *Mpig6b*^*−/−*^ mice (Fig. 3h). Since splenectomy did not alter the established osteosclerosis in the femur of *Mpig6b*^*−/−*^ mice, we hypothesized that splenectomy differentially affects bone resorption, depending on bone size and anatomical location.

**Fig. 4:**
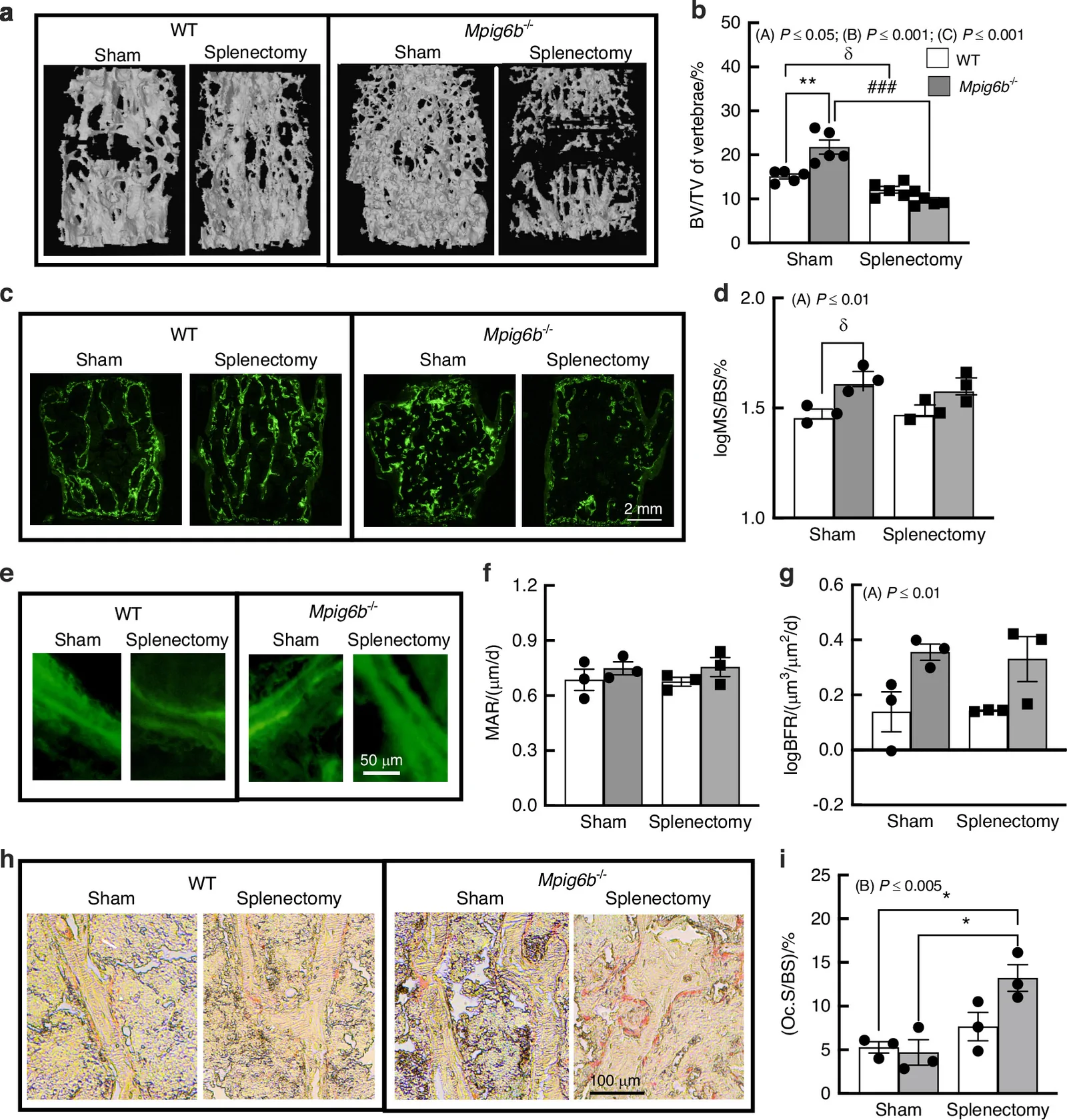
Effect of splenectomy on lumbar vertebrae in *Mpig6b*^−/−^ mice. Splenectomy or sham operations were performed in 32-week-old female WT (white) and *Mpig6b*^*−/−*^ (gray) mice, and lumbar vertebras were characterized 20 weeks later. **a** Representative μCT reconstructions of L3 in WT and *Mpig6b*^*−/−*^ mice in the sham and splenectomy group at the age of 52 weeks (20-week post-surgery). Bone microarchitecture was examined by μCT, shown is **b** bone volume per tissue volume (BV/TV, %). **c**–**g** Double calcein labeling was used to assess bone formation. Representative images of **c** overall calcein labels in trabecular bone of L3 lumbar vertebra, and **e** double calcein lines, scale bars apply to all images in the respective panel. Quantification of **d** mineralized surface per bone surface (%), **f** mineral apposition rate (µm/day), and **g** bone formation rate (µm^3^/µm^2^/d). **h** Representative images of TRAP-positive osteoclasts in the trabecular compartment of L3, scale bar applies to all images. **i** Quantification of osteoclast surface/bone surface (Oc.S/BS). *n* = 3–6 mice per group, statistical analysis by two-way ANOVA, main effects: **a** genotype effect, **b** surgery effect, and **c** interactions. Bonferroni multiple comparisons: *P < 0.05 and **P < 0.01 for comparing genotypes in the same surgery group and ^###^*P* < 0.001 for comparing surgery groups in *Mpig6b*^*−/−*^mice. Unpaired Student’s *t*-test was performed according to the significant effect of surgery reported in BV/TV (**b**) and the effect of genotype reported in MS/BS (**d**); ^δ^*P* < 0.05

We previously showed that lumbar vertebrae were unaffected in 32-week-old female *Mpig6b*^*−/−*^ mice.^21^ We now examined osteosclerosis progression in L3 lumbar vertebra of sham-operated and splenectomized *Mpig6b*^*−/−*^ and WT mice (Fig. 4). In 52-week-old sham-operated *Mpig6b*^*−/−*^ mice, osteosclerosis phenotype was evident in L3 vertebra (Fig. 4a), with higher BV/TV (Fig. 4b) and trabecular number (Fig. S1A) but lower trabecular separation (Fig. S1B) than in sham WT mice. Splenectomy in *Mpig6b*^*−/−*^ mice led to significantly decreased vertebral BV/TV (Fig. 4b). Trabecular thickness was unaffected by splenectomy (Fig. S1C), while the trabecular number decreased (Fig. S1A) and trabecular separation was increased (Fig. S1B) compared to the sham group. In WT mice, splenectomy led to a slight decrease in vertebral BV/TV without altering other parameters of bone microarchitecture (Fig. 4b, Fig. S1A–C). Thus, splenectomy prevented the development of osteosclerosis in the lumbar vertebrae of *Mpig6b*^*−/−*^ mice.

Although we observed no change in plasma levels of bone formation marker P1NP (Fig. 3g), it is still possible that splenectomy locally affects bone formation in vertebrae. Using double calcein labeling, we analyzed bone formation parameters in the vertebrae of 52-week-old *Mpig6b*^*−/−*^ mice (Fig. 4c–g). Overall bone formation was higher in sham *Mpig6b*^*−/−*^ compared to WT mice, and mineralized surface per bone surface remained elevated in splenectomized *Mpig6b*^*−/−*^ mice (MS/BS; Fig. 4d). Consistent with previous observations,^21^ we observed no differences in mineral apposition rate (MAR) between sham or splenectomized *Mpig6b*^*−/−*^ and WT mice (Fig. 4e, f); however, bone formation rate (BFR; Fig. 4g) demonstrated a significant genotype effect, with higher levels in both sham and splenectomized *Mpig6b*^*−/−*^ mice compared to WT mice. We next assessed osteoclast indices on TRAP-stained vertebral bone samples, and identified significantly higher osteoclast surface per bone surface (Oc.S/BS) at L3 in splenectomized *Mpig6b*^*−/−*^ mice compared to their sham-operated and WT groups (Fig. 4h, i). Together with the increased bone resorption marker TRACP 5b (Fig. 3h), these findings suggested that the osteosclerosis-protective effect of splenectomy was mediated by upregulation of osteoclast formation.

**Fig. 5:**
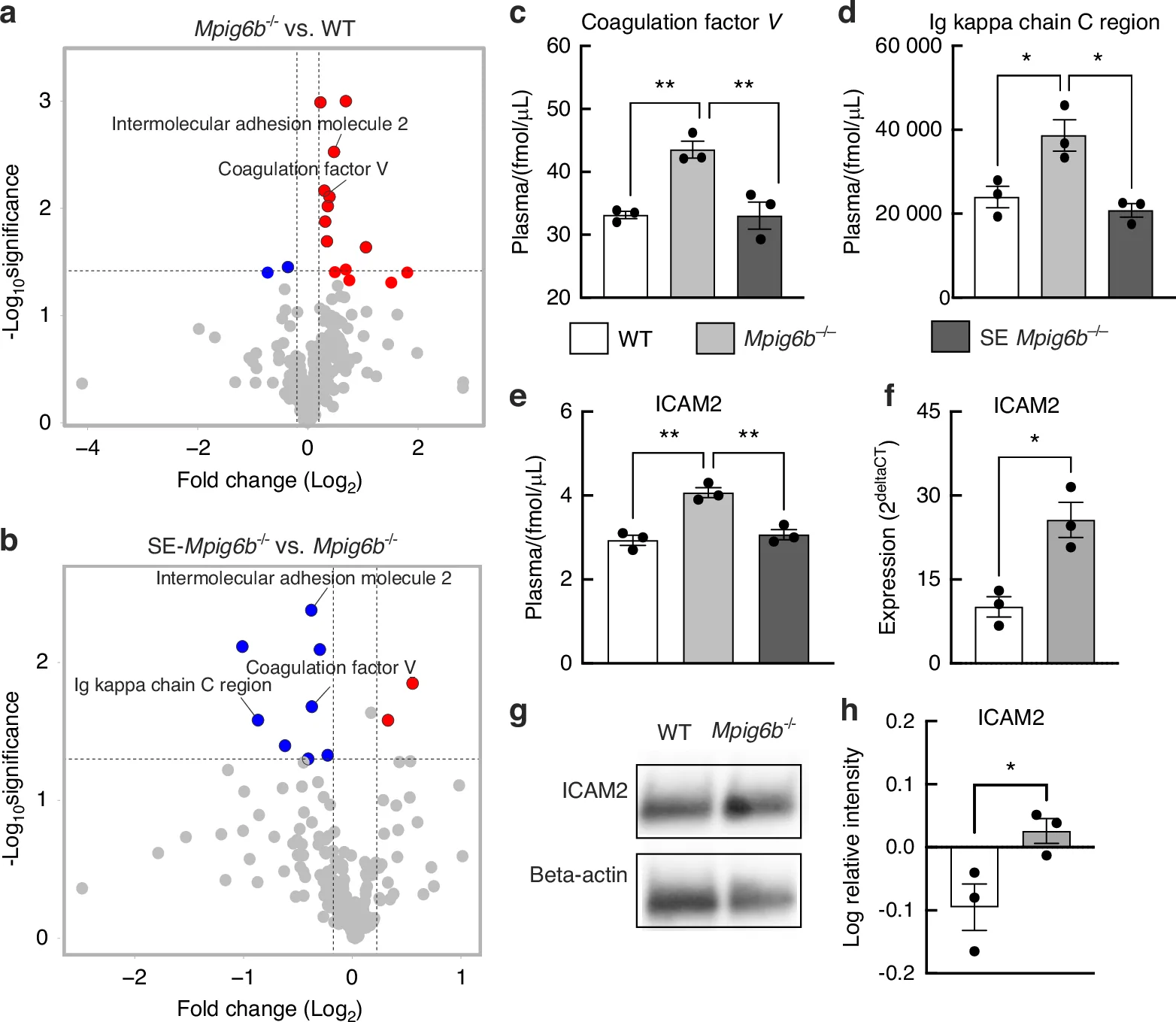
Plasma proteomic study identifies potential mediators affected by splenectomy in *Mpig6b*^−/−^ mice. **a**–**e** Plasma was collected from female 52-week-old WT (white), *Mpig6b*^−/−^ (light gray), and splenectomized *Mpig6b*^−/−^ (dark gray) mice, and plasma proteomics was performed. Volcano plots indicate altered proteins in **a** *Mpig6b*^−/−^ mice compared to WT mice, and **b** in splenectomized compared to *Mpig6b*^−/−^ mice. Gray, blue, and red dots represent the unchanged, decreased, and increased proteins, respectively. Average levels of **c** coagulation factor, **d** Ig Kappa chain C region, and **e** ICAM2 in WT, *Mpig6b*^−/−^ and splenectomized *Mpig6b*^−/−^ mice. *n* = 3 mice per group, one-way ANOVA with Bonferroni multiple comparisons, *P < 0.05 and **P < 0.01. Expression of ICAM2 in the spleens of female 32-week-old WT (white) and *Mpig6b*^−/−^ (gray) mice by **f** quantitative RT-PCR, **g**, **h** immunoblotting showing **g** representative blots and **h** quantification of relative intensity (log scale), beta-actin was used as a loading control. *n* = 3 per group, *P < 0.05 by Student’s *t*-test

We hypothesized that abnormal splenic cells in *Mpig6b*^*−/−*^ mice secrete circulating factor(s) with osteoclast-inhibitory capacity, and that splenectomy reduces circulating levels of these factors. Therefore, we aimed to identify the set of plasma proteins upregulated in *Mpig6b*^*−/−*^ compared to WT mice and normalized following splenectomy. Targeted proteomic analysis^32^ demonstrated several plasma proteins that were significantly higher (red dot) or lower (blue dot) in the *Mpig6b*^*−/−*^ compared to WT mice (Fig. 5a) and in the splenectomized *Mpig6b*^*−/−*^ compared to *Mpig6b*^*−/−*^ mice (Fig. 5b). From those proteins, plasma levels of coagulation factor V (Fig. 5c), Ig Kappa chain C region (Fig. 5d), and ICAM2 (Fig. 5e) were significantly increased in *Mpig6b*^*−/−*^ compared to WT and normalized by splenectomy. Among the 3 proteins, ICAM2 was the only protein previously associated with osteoclastogenesis.^33^ To confirm that circulating ICAM2 originated from abnormal spleens of *Mpig6b*^*−/−*^ mice, we assessed ICAM2 expression in spleens of *Mpig6b*^*−/−*^ mice by qRT-PCR and immunoblotting. The expression of ICAM2 mRNA (Fig. 5f) and protein (Fig. 5g, h) was significantly increased in the spleens of *Mpig6b*^*−/−*^ compared to WT mice. Thus, we identify ICAM2 as a potential osteoclast-regulatory factor secreted by abnormal splenic cells in *Mpig6b*^*−/−*^ mice.

**Fig. 6:**
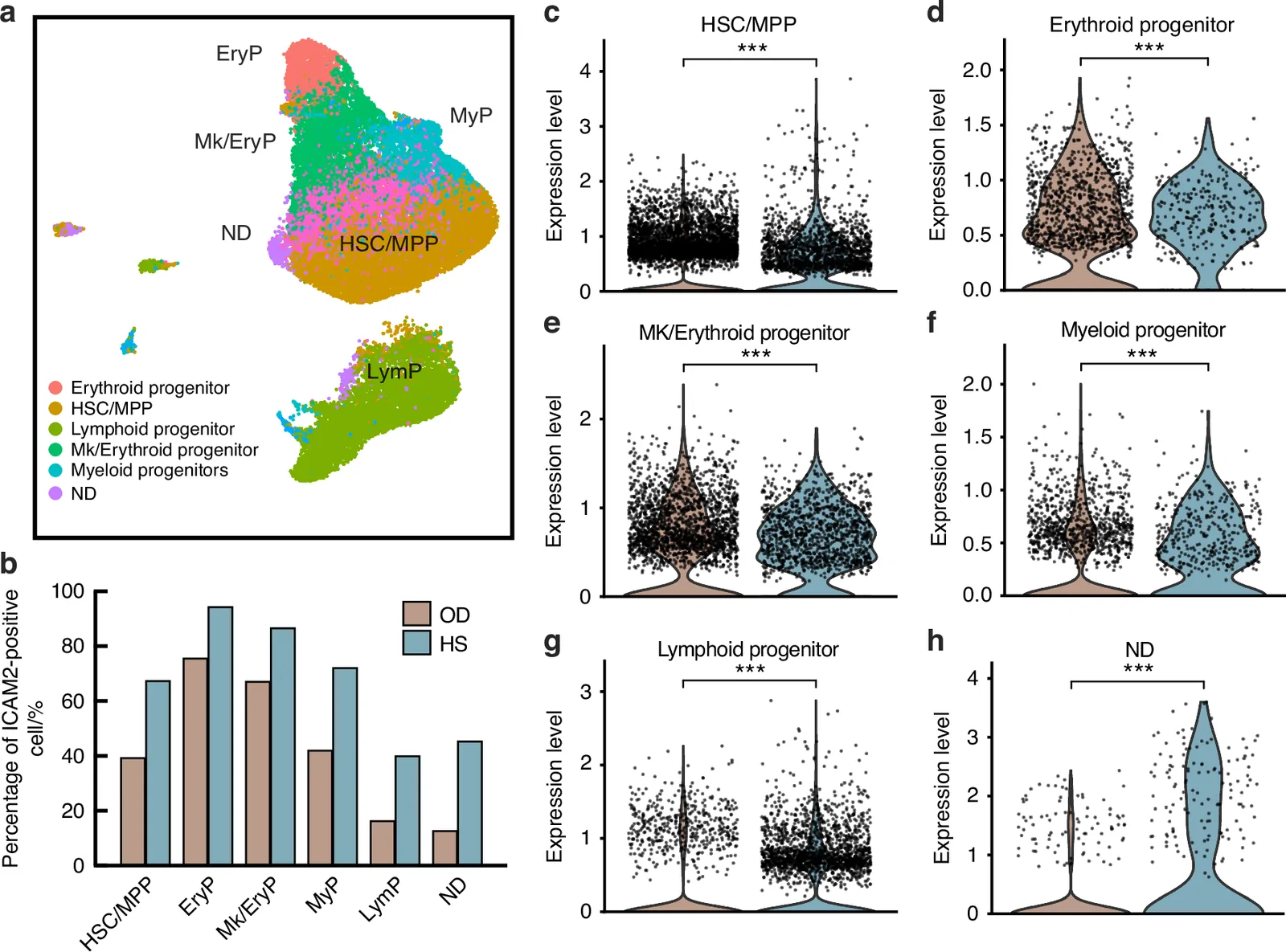
ICAM2 expression in scRNA-seq analysis of spleen from patients with splenomegaly. **a**–**h** Single-cell RNA-seq datasets (count matrices and cell annotations) were obtained from Mende et al.^34^ (GEO accession numbers GSE190067), presenting splenic cells isolated from patients with hereditary spherocytosis (HS; *n* = 2) and deceased organ donors (OD; *n* = 3). **a** cluster of splenic cell population identified in Mende’s dataset. **b** Percentage of ICAM2-positive cells in each cluster. **c**–**h** Violin plots of ICAM2 expression in individual cells of HSC/MPP (**c**), erythroid progenitor (**d**), and MK/erythroid progenitor (**e**). The plots were produced using Seurat’s VlnPlot2 function, and statistical significance was assessed using the Wilcoxon rank-sum test. Cells were considered ICAM2-positive if they had detectable (> 0) ICAM2 counts

To identify potential cellular sources of soluble ICAM2 secreted from abnormal spleens, we performed bioinformatics analysis of published scRNA-seq data from clinical studies. We searched PubMed and the NCBI Gene Expression Omnibus and identified a spleen scRNA-seq dataset (GSE190067) from patients with hereditary spherocytosis (HS) who underwent splenectomy. These patients exhibited splenomegaly and extramedullary hematopoiesis. The dataset was compared to spleen scRNA-seq data from deceased organ donors (OD).^34^ We focused on the specific question: which cells express ICAM2 in the spleen environment, and how does the expression of ICAM2 change with disease progression. In the spleens of HS patients, we focused on 6 clusters of splenic cells: hematopoietic stem cells/multipotent progenitors (HSC/MPP), erythroid progenitors (EryP), megakaryocytes/erythroid progenitors (Mk/EryP), lymphoid progenitors (LymP), myeloid progenitors (MyP), and a non-identified cluster (ND) likely comprising stromal cells (Fig. 6a). Firstly, we assessed the percentage of cells expressing ICAM2 in different clusters. All cell types demonstrated a higher percentage of cells expressing ICAM2 in HS spleen compared to OD; however, the change in percentage of cells expressing ICAM2 was most evident for HSC/MPP, MyP, LymP, and ND (Fig. 6b). When comparing ICAM2 expression levels, we found increased ICAM2 expression in the HS spleen compared to OD in HSC/MPP (Fig. 6c), LymP (Fig. 6g), and ND (Fig. 6h) clusters. In contrast, lower ICAM2 expression was seen in HS spleen compared to OD in EryP (Fig. 6d), Mk/EryP (Fig. 6e), and MyP (Fig. 6f). Thus, HSC/MPP and LymP demonstrated both increased numbers of cells expressing ICAM2 and elevated ICAM2 levels per cell in HS spleens with elevated extramedullary hematopoiesis. A similar pattern was observed in cells that were not identified as hematopoietic (ND cluster), suggesting that stromal cells may also be responsible for ICAM2 production.

**Fig. 7:**
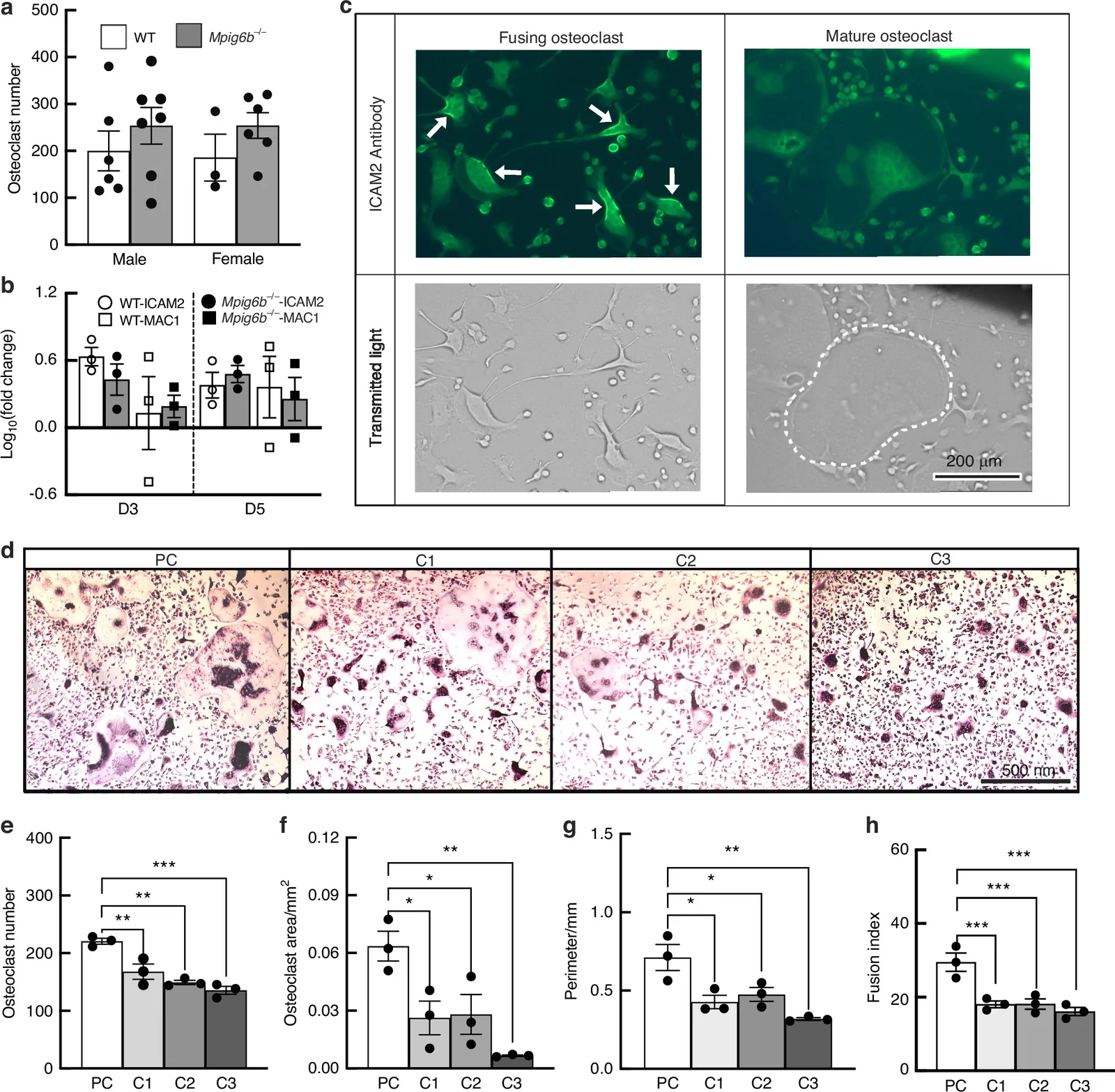
Excessive soluble ICAM2 inhibits osteoclastogenesis. **a**–**d** Bone marrow was isolated from WT (white) and *Mpig6b*^−/−^ (gray) mice, and osteoclast formation in the presence of MCSF and RANKL was examined, or RNA was isolated. **a** Average numbers of osteoclasts formed after 5 days in cultures isolated from male and female WT and *Mpig6b*^−/−^ mice, *n* = 3–7 mice per group, no significant difference by two-way ANOVA. **b** Gene expression of *Icam2* and *Mac1* during osteoclastogenesis from female WT and *Mpig6b*^−/−^ mice assessed on day 3 (D3) and day 5 (D5) of culture. *n* = 3–7 mice per group, no significant difference by two-way ANOVA. **c** Localization of ICAM2 on fusing precursors (left) and mature osteoclasts (right) by immunofluorescence (top) and transmitted light (bottom). Scale bars of 200 µm are shown. **d**–**h** Osteoclastogenesis was induced from WT bone marrow cells without further additions (positive control, PC), or in the presence of recombinant ICAM2 at 3 fmol/μL (C1), 10 fmol/μL (C2), or 14 fmol/μL (C3). **d** Representative images of osteoclast morphology in different conditions, scale bars of 500 nm are shown. Average osteoclast numbers (**e**), area (**f**, mm^2^), perimeter (**g**, mm), and fusion index (**h**). *n* = 3 mice per group, one-way ANOVA with Bonferroni multiple comparison, *P < 0.05 and **P < 0.01, ***P < 0.005

Next, we examined the mechanism by which circulating ICAM2 inhibits osteoclasts. ICAM2 is a subtype of the ICAM family required for cell adhesion. It was previously reported that the interaction of membrane-bound ICAM2 with MAC1 on the cell surface of preosteoclasts is important for osteoclast fusion.^33^ First, we assessed in vitro osteoclastogenesis from bone marrow precursors from 8-week-old male and female *Mpig6b*^*−/−*^ and WT mice, which demonstrated no significant difference in osteoclast formation among the groups (Fig. 7a). ICAM2 and MAC1 were expressed at similar levels on fusing osteoclast (D3) and mature osteoclast (D5) from *Mpig6b*^*−/−*^ and WT mice (Fig. 7b). The visualization of ICAM2 using immunofluorescence demonstrated a more prominent signal at the elongated cell membrane of fusing osteoclasts compared to the large multinucleated osteoclasts (Fig. 7c). Since the interaction of membrane-bound ICAM2 and MAC1 was suggested to promote osteoclast fusion, we hypothesized that the excessive soluble ICAM2 may function as a decoy receptor to competitively bind MAC1 and inhibit the fusion of osteoclasts. To test this hypothesis, we examined osteoclastogenesis from normal osteoclast precursors isolated from 8-week-old female WT mice in the presence of recombinant soluble ICAM2 (rICAM2). Osteoclastogenesis was induced by treatment for 5 days with MSCF and RANKL without further additions (positive control) or with vehicle (no difference from positive control, data not shown), or with rICAM2 at concentrations matching those measured in plasma of *Mpig6b*^*−/−*^ mice: 3 fmol/μL (C1), 10 fmol/μL (C2), or 14 fmol/μL (C3). Osteoclast number, cell size, and fusion index were assessed on day 5 and compared to the positive control group (Fig. 7d). Addition of soluble ICAM2 resulted in a significant dose-dependent decrease in osteoclast number (Fig. 7e), area (Fig. 7f), perimeter (Fig. 7c), and fusion index (Fig. 7h), supporting the role of excessive soluble ICAM2 as an inhibitor of osteoclast fusion.

## Discussion

This study demonstrated the beneficial effect of splenectomy in preventing osteosclerosis in the lumbar vertebrae of female *Mpig6b*^*−/−*^ mice. We identify ICAM2 as a potential osteoclast-regulatory factor secreted from the abnormal spleens of *Mpig6b*^*−/−*^ mice. Our study suggests that excessive soluble ICAM2 is an inhibitor of osteoclast fusion, acting by competitively interfering with the binding of MAC1 and membrane-bound ICAM2, which mediate cell-cell interactions and the fusion of osteoclast precursors. Splenectomy did not adversely affect hematopoietic parameters in female *Mpig6b*^*−/−*^ mice. Since extramedullary hematopoiesis was not observed post-splenectomy, we conclude that hematopoiesis was supported by the bone marrow of osteosclerosis-free vertebral bodies. This study demonstrated the interplay between the spleen-bone axis via the spleen-derived circulating ICAM2, which inhibited osteoclast fusion and promoted osteosclerosis in chronic myelofibrosis.

In our study, splenectomy exerted a beneficial effect on normalizing bone mass and microarchitectures only in vertebrae, but not in the long bones of *Mpig6b*^*−/−*^ mice. One explanation for this phenomenon could be the earlier onset of osteosclerosis in the femur compared to vertebrae in *Mpig6b*^*−/−*^ mice. Our longitudinal study of bone phenotype in *Mpig6b*^*−/−*^ mice reported the onset of osteosclerosis in femurs of female *Mpig6b*^*−/−*^ mice at the age of 16 weeks, which progressively worsened, so that by 32 weeks the normal bone marrow space of long bones was fully occupied by trabecular bone. In contrast, the bone phenotype and mass in vertebrae remain normal at 32-week-old female *Mpig6b*^*−/−*^ mice.^21^ We now demonstrate that in untreated female *Mpig6b*^*−/−*^ mice, osteosclerosis progresses in vertebral bodies, which exhibit high bone mass by 52 weeks. Splenectomy did not reverse the established osteosclerosis in the femurs but prevented the onset of osteosclerosis in the lumbar vertebrae of *Mpig6b*^*−/−*^ mice. We propose that alleviation of osteoclast inhibition post-splenectomy is a mechanism for preventing osteosclerosis in *Mpig6b*^*−/−*^ vertebrae, as evidenced by the increased osteoclast surface per bone surface in lumbar vertebra and bone resorption marker (TRAcP 5b) in plasma. It is possible that after osteosclerosis is established in long bones and the volume of bone marrow cells is largely reduced, the frequency of osteoclast precursors, already low in normal bone marrow, is insufficient to reverse osteosclerosis. Osteosclerosis in patients with primary myelofibrosis is commonly reported in the proximal segments of long bones, the vertebral column, or the pelvis.^14,35,36^ Currently, the decision to perform splenectomy in symptomatic MF patients is guided by their hematological presentation.^7,23^ Our study suggests that the degree and location of osteosclerosis may also be used in clinical splenomegaly management decision-making.

Based on these findings, we propose ICAM2 as a potential soluble regulator of bone homeostasis in MF. Relatedly, circulating ICAM2 levels were shown to have prognostic value in several clinical studies. Of relevance to this study, a high level of circulating ICAM2 in patients with idiopathic pulmonary fibrosis was demonstrated to negatively correlate with pulmonary function.^37^ Moreover, circulating ICAM2 levels were increased in patients with myeloid neoplasms and significantly correlated with the hazard ratio, where it is considered a signature plasma protein for predicting Myeloid Neoplasm risk.^38^ While less clinically relevant, the levels of circulating ICAM2 were shown to correlate with clinical outcomes in chronic heart failure patients^39^ also supporting the translational relevance of our findings. ICAMs are cell-surface proteins; however, our study demonstrates significant levels of circulating, soluble ICAM2. In this regard, ICAM5 has been reported to be cleaved and released into the bloodstream in a soluble form by matrix metalloproteinases.^40^ In addition to ICAM2, we found that plasma levels of Ig kappa chain C and coagulation factor 5 were increased in *Mpig6b*^*−/−*^ mice and normalized after splenectomy. Since our bone turnover analysis suggested that splenectomy increases osteoclast function, and ICAM2 has previously been shown to be important for osteoclastogenesis,^33^ we have focused our studies on ICAM2. Nevertheless, the role of Ig kappa chain C and coagulation factor 5 in MF requires further exploration.

Our study identifies the enlarged spleens of female *Mpig6b*^*−/−*^ mice as a source of circulating ICAM2; however, the question of which cells produce ICAM2 remains open. Expression of ICAM2 has been demonstrated on platelets,^41^ and MKs.^42^ MKs have previously been shown to interact with osteoblasts and osteoclasts, regulating bone homeostasis through cell-cell interactions and multiple soluble factors.^11,15^ While myelofibrotic splenic MKs may be a source of ICAM2, the in-depth single-cell analysis of MKs in patients with MF did not identify ICAM2 as a factor associated with abnormal MK count or function.^43^ To assess the potential contribution of other hematopoietic or stromal cells in ICAM2 production, we performed bioinformatics analysis of splenic scRNA-seq datasets from patients with HS undergoing splenectomy due to extramedullary hematopoiesis and splenomegaly.^34^ HSC/MPP and lymphoid lineage cells demonstrated high ICAM2 expression in the abnormal spleens of HS patients, while cells of erythroid/megakaryocytic origin demonstrated lower/unchanged ICAM2 expression. The ICAM2 expression by epithelial and endothelial cells was shown to be important in inflammatory conditions.^44,45^ Since systemic and local inflammation has been suggested to be a significant part of the pathophysiology of MF,^46,47^ further studies are required to identify the source of ICAM2 production in MF.

We demonstrated the mechanism by which soluble ICAM2 may inhibit osteoclast function in *Mpig6b*^*−/−*^ mice. A previous study demonstrated the critical role of cell-surface-bound ICAM2 in osteoclast fusion, mediated through binding of ICAM2 to its cognate receptor MAC1 on the cell surface of the neighboring osteoclast precursors.^33^ We propose that, in contrast to surface-bound ICAM2, soluble ICAM2 acts as a decoy receptor by competitively binding to MAC1, leading to reduced interactions between membrane-bound ICAM2 and MAC1, thus suppressing osteoclast fusion. This idea is directly supported by our in vitro study, which demonstrates that recombinant ICAM2 supplementation dose-dependently inhibits osteoclast fusion. This action of soluble ICAM2 is similar to the interactions among three well-described osteoclast modulatory cytokines: the receptor RANK, its ligand RANKL, which is physiologically expressed on the cell surface, and the soluble decoy receptor osteoprotegerin (OPG), which binds to RANKL, preventing it from interacting with RANK.^48–51^ Our study suggests that the increased systemic soluble ICAM2 mediates osteoclast inhibition and supports osteosclerosis in *Mpig6b*^*−/−*^ mice.

Our study has several limitations. While our study strongly suggests that the spleen is the source of increased soluble ICAM2 in the plasma of the *Mpig6b*^*−/−*^ mice, it remains unclear which specific splenic cells secrete ICAM2. Our studies are limited to female *Mpig6b*^*−/−*^ mice. Designing the studies that directly compare the effect of splenectomy in male and female *Mpig6b*^*−/−*^ mice is challenging, because we previously showed a more rapid onset of progressive osteosclerosis in female *Mpig6b*^*−/−*^ mice compared with their male counterparts.^21^ In male *Mpig6b*^*−/−*^ mice, spleen size was normal at 32 weeks, and may become enlarged only in older mice. Thus, experimentally matching the age of male and female *Mpig6b*^*−/−*^ animals starts with different presentations (splenomegaly in females, normal spleen size in males), while experimentally matching the presentation of a similar degree of splenomegaly will need to be performed in mice of dramatically different ages. Therefore, the data obtained in female mice in our study are likely not applicable to male mice.

Taken together, our study demonstrates the potential role of soluble ICAM2 as a mediator in the spleen-bone axis in G6b-B-deficiency and other clinical hematological disorders. This finding suggests that membrane-bound ICAM2 may be a potential drug target, while soluble ICAM2 may be a potential treatment for conditions associated with altered bone homeostasis, in particular MF-associated osteosclerosis. Moreover, circulating levels of ICAM2 should be explored for early detection and/or as a prognostic marker of splenomegaly and osteosclerosis. Given the beneficial effect of splenectomy on preventing osteosclerosis observed in our study, the benefits of considering bone homeostasis alongside hematological parameters when deciding to perform splenectomy should be explored. Thus, our translational study opens new avenues for the diagnosis and treatment of osteosclerosis associated with MF.

## Materials and methods

### Animals

Constitutive *Mpig6b* homozygous knockout (*Mpig6b*^*−/−*^) and wildtype (WT, *Mpig6*^*+/+*^ mice)^18^ on a C57BL/6J background were maintained by crossing mice heterozygous for the *Mpig6b* null mutation (*Mpig6*^*+/−*^) in a pathogen-free standard animal facility at the Shriners Hospital for Children—Canada. All animals had unrestricted access to food and water and were on a 12-h alternating light and dark cycle. All experiments were approved by the Shriners Facility Animal Care Committee (AUP-SHRN-7127) and confirmed by the ethical guidelines of the Canadian Council on Animal Care.

### Splenectomy surgery

Litters of WT and *Mpig6b*^*−/−*^ mice were randomly assigned to either splenectomy or a sham operation. Surgeries were conducted at 32 weeks of age. Splenectomy surgeries were performed at the Shriners Hospital for Children—Canada, according to standard operating procedures, according to SOP 3.9 Rodent splenectomy. In brief, animals were administered 20 mg/kg Rimadyl (Pfizer, New York, NY, USA) and were anesthetized with isoflurane (induction: O2, 1 L/min; isoflurane, 5%; maintenance: O2, 1 L/min; isoflurane, 2.5%–3%). Analgesic agents 1% Lidocaine (Wyeth, Saint-Laurent, Canada) and 0.25% Bupivacaine (Abbott Laboratories Ltd., Edmonton, Canada) were applied to the surgical site just prior to making an incision. Splenectomy surgery was performed by making a midline incision, separating the skin from the underlying muscle, and then making an incision in the spleen. Sham operation included the same steps of the splenectomy surgeries, but the spleen was not removed.

### Hematopoietic parameters analysis

Animals were euthanized at 52 weeks of age. Whole blood was collected in vials containing 2.0–2.5 mg/mL K_3_EDTA. Following blood collection, the vials were gently centrifuged at 1 000 × *g* for 10 min, and plasma was separated from the RBCs, and aliquots were frozen at −80 °C. Hematological parameters (platelet counts, platelet size, hematocrit, RBC count, MCV, and RBC distribution width) in the peripheral blood of WT and *Mpig6b*^*−/−*^ mice were evaluated as described by Mazharian and colleagues.^18^ In addition, hematopoietic organs, namely liver, kidney, and lung, were isolated from the mice at the date of euthanasia for measuring organ weight.

### Histological analysis of the spleen

The spleen was isolated, weighed, and measured at the time of splenectomy at the age of 32 weeks. The spleens were fixed in 4% paraformaldehyde, dehydrated by an ethanol gradient, and stored in 70% ethanol at 4 °C before paraffin embedding. The specimens were longitudinally cut into slices at a thickness of 4 μm, affixed to glass slides, deparaffinized, rehydrated, and subjected to hematoxylin and eosin (H&E) staining. Images were taken using a Leica DMRB light microscope equipped with the Olympus Nanozoomer 2.0-HT System with NDP scan 2.5 image software.^52^ Identification of megakaryocyte density and cell size followed the previously described guidelines^53,54^ using ImageJ software.

### Micro-computed tomography (μCT) and analysis of bone microarchitectures

In vivo μCT scans of femurs of living mice were performed using a SkyScan 1276 scanner (Bruker, Kontich, Belgium) under isoflurane anesthesia (induction: O_2_, 2 L/min; isoflurane, 3%; maintenance: O_2_, 0.9 L/min; isoflurane, 2%) at 32 weeks, right before the surgeries, and at the time of euthanasia 20-week post-surgery. For ex vivo μCT, lumbar vertebrae were isolated at euthanasia and fixed in 10% formalin at 4 °C for 24 h, followed by rinsing in 1× phosphate-buffered saline (PBS) solution, pH 7.4, at 4 °C for 24 h. Ex vivo μCT was performed using the SkyScan 1272 scanner (Bruker, Kontich, Belgium). The scans were reconstructed using Skyscan NRecon software (Bruker Optics Inc., Billerica, MA, USA) with the constant beam hardening correction (20%) and ring artifact corrections (2) applied to all samples. *X*/*Y* alignment correction was applied to the ex vivo scans. Selection of the volume of interest (VOI), automatic segmentation of the region of interest (ROI), and standard three-dimensional morphometric analysis of bone architecture were performed using Skyscan CT analyzer software (Bruker Optics Inc.). Femoral VOI corresponded to 10% of the bone length and was selected as 100 μm below the growth plate. VOI of the third lumbar vertebra corresponded to a region 500 μm from the proximal and 500 μm from the distal endplates. Bone microarchitecture is reported according to the guidelines in Bouxsein and colleagues.^55^ Analyzed parameters were bone volume to tissue volume fraction (BV/TV), cortical thickness (C.Th), trabecular thickness (Tb.Th), trabecular number (Tb.N), trabecular separation (Tb.Sp), and bone marrow area per tissue area (Ma.Ar/T.Ar, %).

### Bone biomechanical analysis

Femurs were isolated at euthanasia at the age of 52 weeks (20 weeks post-surgery) for mechanical testing using a 3-point bending test (3PBT). Briefly, femurs were dissected and wrapped in gauze soaked in PBS solution pH 7.4 and stored at −20 °C. Frozen femurs were equilibrated to room temperature and loaded to failure in a three-point bending assay using an Instron model 5943 single-column table frame machine (Instron, Norwood, MA, USA). Samples were positioned with the anterior surface down and loaded centrally at a crosshead speed of 0.05 mm/s and a support distance of 5 mm. Whole-bone mechanical properties were assessed by the generated force versus displacement data.

### Bone histomorphometric analysis

Fifty-week-old mice received two intraperitoneal injections of 30 μg/g calcein (SIGMA) with a 10-day interval and were euthanized 3 days after the last injection. Lumbar vertebrae L3-5 were fixed for 24 h in 10% phosphate-buffered formalin solution at 4 °C. Undecalcified bones were dehydrated by passing through graded alcohols and embedded in methyl methacrylate (MMA) resin. Bone dynamic histomorphometric evaluation was performed on 6-μm-thick undeplasticized sections. Imaging of calcein double-label was performed with FITC and UV filter sets of a Nikon T2000 fluorescent inverted microscope (Nikon, Tokyo, Japan) and a cooled charge-coupled device camera (Hamamatsu Photonics, Hamamatsu City, Japan) connected to the Volocity image analysis software (Improvision, Coventry, UK). Data are presented according to the ASBMR guidelines,^56^ and the analyzed parameters were MAR, mineralizing surface to bone surface (MS/BS), and BFR. To assess osteoclast indices, first, MMA-embedded bone sections were deplastified using a sequential solvent treatment. Briefly, slides were heated at 55 °C for 15 min and cooled to room temperature, then immersed in fresh 2-methoxyethyl acetate, acetone, 75% ethanol, and 40% ethanol, with a final rinse in water. Slides were then incubated for 20 min at room temperature in sodium acetate buffer pH 5.0 containing 0.2 mol/L sodium acetate (Fisher, 308269), 50 mmol/L L(+) tartaric acid (Sigma, 228729), ddH_2_O, and acetic acid (Fisher, BP2401-212). Next, Fast Red TR salt hemi(zinc chloride; Sigma, 368831) and naphthol AS-MX phosphate (Sigma, N4875) were dissolved in buffer immediately before use, and slides were incubated in staining solution at 37 °C for 1.5 h. Then, the slides were washed with water twice, and light-fast green FCF staining (0.2%; Sigma 7252) was added for 15 s. After the last wash in water, the slides were mounted and dried. Images were taken using a Leica DMRB light microscope equipped with BioQuant Measurement software. The osteoclast surface/bone surface (Oc.S/BS) was assessed at the ROI, measuring 1 600 μm in height and 1 400 μm in width, located approximately at 50 μm from the proximal end of the growth plate and 50 μm away from the cortical bone compartment in all samples. Analysis was performed using ImageJ software.

### ELISA assays

Whole blood samples were collected in vials containing 2.0–2.5 mg/mL K_3_EDTA from mice at the age of 52 weeks. The vials were gently centrifuged at 1 000 × *g* for 10 min for plasma collection. The level of TRACP 5b in plasma was tested on 4× diluted plasma with MouseTRAP™ ELISA (Immunodiagnostic Systems, Boldon Business Park, UK), and the level of P1NP was tested on 10× diluted plasma with Rat/Mouse PINP enzyme immunoassay (EIA) (Immunodiagnostic Systems) according to the manufacturer’s protocols. Absorbance was measured with VICTOR® Nivo™ microplate reader (PerkinElmer, Waltham, MA, USA).

### Proteomics study in plasma

Quantitative proteomic and bioinformatic analyses were performed using the BAK375 mouse kit in one 1290 HPLC method with MRM-based methodology and stable isotope-labeled standards (SIS).^32^ Briefly, 10 μL of plasma from each mouse was subjected to 9 mol/L urea, 20 mmol/L dithiothreitol, and 0.5 mol/L iodoacetamide, sequentially in a Tris buffer at pH 8.0. Denaturation and reduction occurred simultaneously at 37 °C for 30 min, with alkylation occurring thereafter in the dark, at room temperature for 30 min. Proteolysis was initiated by the addition of TPCK-treated trypsin (25 μL at 1 mg/mL; Worthington) at a 20:1 substrate: enzyme ratio and incubated at 37 °C overnight. Proteolysis was quenched with formic acid (FA), and the SIS peptide mixture was then spiked into the samples. All samples were then concentrated by solid-phase extraction (Oasis HLB, 2 mg sorbent; Waters), evaporated, and rehydrated in 0.1% FA to a final concentration of 1 μg peptides/μL digest for LC-MRM/MS.

### Bioinformatics study of single-cell RNA-sequencing (scRNA-seq) data

Single-cell RNA-seq datasets (count matrices and cell annotations) were obtained from Mende et al.^34^ (GEO accession numbers GSE190067). Count matrices (genes × cells) were loaded into the R package Seurat^57^ for downstream analyses. Following SCTransform normalization, individual samples were integrated using the RPCAIntegration procedure. UMAP dimension reduction was generated based on the first 13 principal components. Cell types were assigned based on the annotations provided in the original publication. Cells without assigned annotations were excluded from downstream analyses. Violin plots of ICAM2 expression were produced using Seurat’s VlnPlot2 function, and statistical significance was assessed using the Wilcoxon rank-sum test. Cells were considered ICAM2-positive if they had detectable (> 0) ICAM2 counts, and the percentage of expressing cells was computed separately for each cell type.

### Primary bone cell isolation and osteoclastogenesis

Mouse bone marrow cells were collected as previously described.^58^ Mouse bone marrow cells were collected from 8-week-old *Mpig6b*^*−/−*^ mice and their littermate WT. Cells were cultured in 75 cm^2^ tissue culture flasks (~1.5 × 10^7^ cells per flask) with human recombinant macrophage-colony stimulating factor (M-CSF, 25 ng/mL, 300–25, PeproTech Inc.) for 24 h overnight. The next day, the non-adherent cells were collected and plated at 5 × 10^4^ cells/cm^2^ in α-MEM medium supplemented with 100 units/mL penicillin, 100 µg/mL streptomycin, and 10% fetal bovine serum, M-CSF (50 ng/mL), and recombinant GST-RANKL (50 ng/mL). Medium was changed every other day and culture for 5–6 days to achieve mature osteoclasts in the culture. To study the effect of excessive ICAM2 on osteoclastogenesis, the recombinant ICAM2 (Mouse, Recombinant hFc Tag, Sinobiological, NP_034624.1) was reconstituted in sterile PBS pH 7.4 and directly added to the working cultured media of osteoclast at day 1 and day 3 of the culture with final concentrations of 3 fmol/μL, 10 fmol/μL, and 14 fmol/μL as treatment groups. The sterile PBS pH 7.4 was used as a control for treatment. On day 5–6, cells were fixed using 10% formalin (23-245-685, Fisher) and stained for (i) tartrate-resistant acid phosphatase (TRAP, Sigma-Aldrich Co, 387A-KT) and (ii) immunofluorescence staining for ICAM2 (rat IgG2a kappa Isotype Control Alexa Fluor™ 488, eBioscience™, 53102182). Osteoclasts were identified as multinucleated (> 3 nuclei). TRAP-positive cells were further characterized by image analysis using PixeLINK Capture SE® software (PixeLINK) and ImageJ.

### Gene expression in osteoclasts and spleen

Upon euthanasia, the spleen was directly snap frozen in liquid nitrogen and stored at -80 °C. The spleens were cut into 2 mm × 2 mm and placed in TRI reagent® (Sigma, Kanagawa, Japan) and mechanically homogenized (Omni TH; Omni International, Kennesaw, GA, USA). RNA from the spleen was extracted following the TRI reagent® protocol. RNA was isolated from osteoclast culture at days 1, 3, and 5 using the RNeasy Mini Kit (Qiagen, 74104). The first-strand cDNA synthesis was performed using a high-capacity cDNA reverse transcription kit (Applied Biosystems, Foster City, CA, USA), followed by gene expression analysis performed on duplicates using a quantitative real-time PCR (qRT-PCR) system (Model 7500; Applied Biosystems, Foster City, CA, USA) and SYBR® Green PCR Master Mix (Applied Biosystems, Carlsbad, CA, USA). Glyceraldehyde 3-phosphate dehydrogenase **(***Gapdh*, NM_001289726.1) was used as the reference gene with the forward primer: 5′ ACCCAGAAGACTGTGGATGG 3′ and the reverse primer: 5′ CACATTGGGGGTAGGAACAC 3′. The geometric mean of their threshold values was used for further calculations. To study ICAM2 expression (NM_001082960), we used forward primer: 5′ TATGAGCCGATGCAGGACAAC3′ and reverse primer: 5′ GTGCTGGCCAAAGATAAAGCATAG 3′. For MAC1 expression (NM_001082960), we used forward primer: 5′ AAAGCCATTGTGGCCAGT 3′ and reverse primer: 5′ GTCATCTCTGAAGCCGTGA 3′. The relative fold expression was calculated by the delta cycle threshold method.^59^

### Western blot analysis

The homogenized spleen samples in TRI reagent® (Sigma, Kanagawa, Japan) were processed as described previously.^60^ Briefly, the protein lysate was lysed using a RIPA buffer (50 mmol/L Tris-HCl, pH 7.4, 150 mmol/L NaCl, 1% Nonidet P-40, 1 mmol/L EDTA, 10 mg/mL aprotinin, 1 mg/mL leupeptin, 0.1 mol/L phenylmethylsulfonyl fluoride, 0.5 mol/L sodium fluoride, and 1 mmol/L sodium orthovanadate). The protein concentrations were determined (Quant-iT™ Protein Assay Kit, Invitrogen, Q33210). Proteins were re-dissolved in SDS sample buffer, separated by 7.5% gel electrophoresis under reducing conditions, transferred to a nitrocellulose membrane (Bio-Rad, #162-0115; 0.45 mm) using 10 mmol/L sodium tetraborate, blocked in 5% non-fat milk in TBST buffer (10 mmol/L Tris-HCl, pH 7.5, 150 mmol/L NaCl, 0.05% Tween 20) for 1 h at 37 °C followed by overnight incubation at 4 °C with the primary antibodies: mouse ICAM2/CD102 Antibody (RNDsystems, F774-SP, 1:1 000) and β-Actin (8H10D10) Mouse antibody (Cell Signaling, 3700, 1:1 000), then incubated with horseradish peroxidase-conjugated secondary antibodies NeutrAvidin (Thermo Scientific, 31001, 1:800) and visualized using a chemiluminescence substrate (Thermo Scientific, 32106).

### Statistical analysis

All analysis was performed on age-matched littermates by two-way ANOVA with Bonferroni posttest considering the effect of genotype, surgery, and interaction, and a Student’s *t*-test in GraphPad Prism 9 software (GraphPad Software, Inc., La Jolla, CA, USA) at 95% significance level with a two-tailed distribution. All data are presented as means with SEM and the number of mice (*n*) per group.

## Supplementary information

Supplemental Figure
