# Supplementary material for: Spleen-derived circulating intercellular adhesion molecule 2 promotes osteosclerosis in chronic myelofibrosis
Source: Bone Res. 2026 Sep 20;14:100. doi: 10.1038/s41413-026-00575-9 (PMC13590502; doi:10.1038/s41413-026-00575-9)
Supplement: Supplementary file 1 [file 41413_2026_575_MOESM1_ESM.pdf]

Supplementary material for the manuscript

Title: Spleen-derived circulating intercellular adhesion molecule 2 promotes osteosclerosis in chronic myelofibrosis

Authors: Sirion Aksornthong, Kerstin Tiedemann, Mélanie Welman, Alexandra Mazharian, Marie Lordkipanidzé, Yotis A Senis, Svetlana V. Komarova

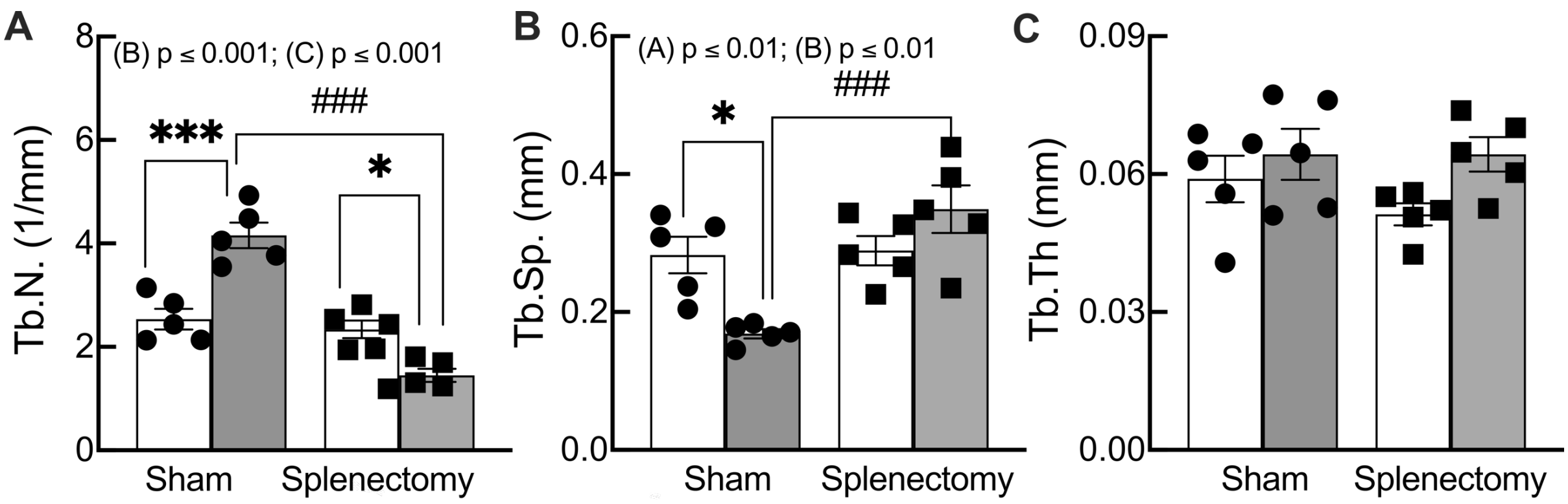

Supplementary figure S1. Effect of splenectomy on lumbar vertebra in *Mpig6b*$^{-/-}$ mice. Splenectomy or sham operations were performed in 32-week-old female WT (white) and *Mpig6b*$^{-/-}$ (grey) mice, and lumbar vertebras were characterized at the age of 52 weeks (20-week post-surgery). (A-C) Bone microarchitecture of the trabecular compartment of L3 in WT and *Mpig6b*$^{-/-}$ mice in the sham and splenectomy group at the age of 52 weeks (20-week post-surgery) was examined by μCT, shown are (A) trabecular number (Tb.N, 1/mm), (B) trabecular separation (Tb.Sp., mm) and (C) trabecular thickness (Tb.Th, mm). n=5 mice per group. Statistical analysis by two-way ANOVA, main effects: (A) genotype effect, (B) surgery effect, (C) interactions. Bonferroni multiple comparisons: *$p < 0.05$, ***$p < 0.001$ compared genotypes within the same surgery group; $^{###}p < 0.001$ compared surgery groups within *Mpig6b*$^{-/-}$ mice.
